# Partial splenectomy for massive malarial splenomegaly: A unique case from an Australian regional centre

**DOI:** 10.1016/j.ijscr.2025.111036

**Published:** 2025-02-07

**Authors:** Minella Lalloz, Kate Swift, Omar Mouline, Christian Beardsley

**Affiliations:** a The General Surgery Department at the Cairns Hospital, 165 The Esplanade, Cairns, QLD 4870, Australia; b The James Cook University, 1/14-88 McGregor Rd, Smithfield, QLD 4878, Australia

**Keywords:** Partial splenectomy, Hyperreactive malarial splenomegaly syndrome, Tropical splenomegaly syndrome, Malaria, *Plasmodium falciparum*

## Abstract

**Introduction and importance:**

This report describes a rare and remarkable case of partial splenectomy (PS) performed to manage the sequelae of massive malarial splenomegaly. It is likely the first reported case to date.

**Case presentation:**

A 40-year-old female from Papua New Guinea presented shocked to a remote hospital in the Torres Strait Islands. She had a history of hyperreactive malarial splenomegaly syndrome (HMSS) during childhood. After urgent transfer and stabilisation in a regional centre, the patient underwent a PS.

**Clinical discussion:**

Indications for surgery included hypersplenism, acute on chronic splenic infarction, portal vein thrombosis with portal hypertension and varices, and recurrent gastrointestinal bleeding. The decision to operate was complex, and perioperative optimisation required a multidisciplinary team.

**Conclusion:**

This report adds new and valuable information to the current literature on the indications for PS. Moreover, it reminds clinicians about massive splenomegaly from *P. falciparum* malaria, associated sequelae, and the diagnostic and therapeutic challenges which are seldom encountered in Australia today.

## Highlights

• This report describes a unique case of partial splenectomy (PS) performed to manage the sequelae of massive malarial splenomegaly in a female from Papua New Guinea.
• Formerly known as tropical splenomegaly syndrome (TSS), hyperreactive malarial splenomegaly syndrome (HMSS) is defined as massive splenic enlargement caused by an abnormal immune response, namely chronic antigenic stimulation to malarial parasites.
• Currently, there are no evidence-based decision-making guidelines when considering PS for hypersplenism and previous HMSS.
• Each individual case should be carefully selected and the decision for PS made in conjunction with a multidisciplinary team of infectious disease specialists, haematologists, gastroenterologists, and expert surgeons.

## 1 Introduction

Hyperreactive malarial splenomegaly syndrome (HMSS) is defined as massive splenic enlargement caused by an abnormal immune response, namely chronic antigenic stimulation to malarial parasites [1]. Formerly known as tropical splenomegaly syndrome (TSS), HMSS is the leading cause of splenomegaly in malaria-endemic areas, including sub-Saharan Africa, Southeast Asia, Central and South America, and the South Pacific [[1], [2], [3]]. According to Fakunle's criteria proposed in 1981 [4], the diagnosis of HMSS can be made based on clinical and laboratory testing parameters combined with epidemiological context (i.e., living or travelling within malaria-endemic areas).

Malaria is a highly transmissible disease that spreads through the bite of infected female *Anopheles* mosquitoes. There are five *Plasmodium* parasite species [5], with *P. falciparum* accounting for 90 % of the world's malaria mortality [6]. Chronic exposure to *P. falciparum* can lead to massive splenomegaly and HMSS, which can be fatal if left untreated. Severe malaria is rarely observed in Australia. Surgical management of malarial splenomegaly is even more uncommon and often not recommended in the literature [7,8].

Massive splenomegaly is defined as an enlarged spleen measuring greater than 20 cm in length. Causes of splenomegaly may be infectious (malaria, tuberculosis, human immunodeficiency virus, schistosomiasis, leishmaniasis, mononucleosis), malignant (chronic myeloid leukaemia, primary splenic lymphoma), hepatic (cirrhosis, hepatitis), haematological (haemolytic anaemia) or thrombotic (portal or splenic vein thrombosis) [[9], [10], [11], [12], [13]].

In Australia, the incidence of massive splenomegaly secondary to *P. falciparum* is extremely uncommon, especially since malaria was declared eradicated from the country in 1981 [14]. The few reported cases are in individuals who have travelled to or immigrated from malaria-endemic regions. Performing a total splenectomy for massive splenomegaly is even rarer, making this case of partial splenectomy (PS) – which is a high risk and technically more challenging operation – unique.

## 2 Case presentation

### 2.1 The case presentation

A 40-year-old female from Papua New Guinea presented acutely unwell to a remote hospital in the Torres Strait Islands. She had dual pathology - necrotising *Streptococcal* pneumonia requiring antibiotics and intercostal catheter insertion, and upper gastrointestinal bleeding (UGIB) with severe anaemia. After stabilisation locally, she was subsequently transferred to a regional centre in Australia. She had a history of HMSS in childhood, chronic pancytopenia, multiple splenic infarcts, portal vein thrombosis, and portal hypertension with sinistral varices. Her only medication was oral propranolol (10 mg twice daily). Past surgical history was significant for an emergency oesophagogastrectomy through a rooftop incision for uncontrolled variceal bleeding aged 10-years-old. She had no other medical history and was a non-smoker and non-drinker.

### 2.2 The work up

On clinical examination, she appeared unwell and cachectic, with scleral icterus. Chest auscultation revealed coarse global crepitations. Her abdomen was soft and non-tender, with a palpable spleen in the left lower quadrant. There were no ascites or stigmata of liver disease. Her body mass index (BMI) was 14.9 kg/m^2^, based on a weight of 38.2 kg and height of 160 cm.

Her initial haemoglobin level of 32 g/L improved to 62 g/L after 4 units of packed red blood cells (PRBC). She had an associated iron deficiency and microcytosis (mean cell volume 62 fl). She also had thrombocytopenia (platelets 68 × 10^9^/L), coagulopathy (international normalised ratio 1.6), and hypoalbuminemia (albumin 11 g/L). Serial liver function tests revealed hyperbilirubinemia (up to 120 μmol/L), with minimal liver enzyme derangement.

**Fig. 1:**
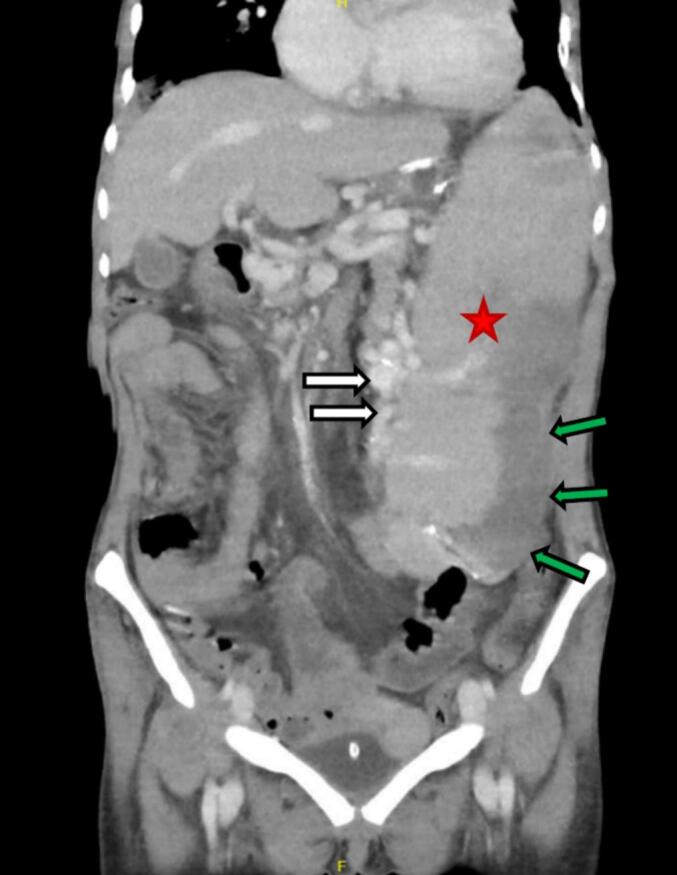
Pre-operative CT of the abdomen and pelvis demonstrating massive splenomegaly (red star) with associated splenic hilar and lienorenal varices (white arrows) and infarcted inferolateral area of the spleen (green arrows). Patient has a history of malaria and left-sided portal hypertension with varices.

Contrast-enhanced computed tomography (CT) showed massive splenomegaly, measuring 25 cm in length, with a large infarct in the latero-inferior segments. Multiple splenic hilar and lienorenal varices were observed (Fig. 1). The portal vein appeared patent with peripheral calcifications, likely to represent previous vein thrombosis. Chest imaging was consistent with the right-sided multilobar pneumonia.

The patient was initially admitted to the respiratory team for necrotising *Streptococcal* pneumonia. A gastroenterology opinion was promptly obtained to investigate her anaemia, and upper gastrointestinal endoscopy revealed bleeding from portal hypertensive gastropathy and known oesophageal varices with no stigmata of recent bleeding. A haematological opinion was also sought in the context of pancytopaenia; both the haemolysis screen and bone marrow aspiration and trephine (BMAT) biopsy were unremarkable. Serological test results for human immunodeficiency virus, hepatitis B, and hepatitis C were negative. IgM levels were normal. Of note, the malarial antibody test was not performed as it is no longer available in Australia, owing to the rarity of HMSS as a diagnosis.

### 2.3 Request for surgical consultation

While splenomegaly was longstanding in this patient, there was clear decompensation in clinical status, with an ongoing need for regular blood products for severe anaemia and bleeding secondary to portal hypertensive gastropathy. Hence, a surgical consultation was requested for consideration of splenectomy, noting the high risk of morbidity and mortality both in the short and long term. Given that the patient would return to a malaria-endemic region, asplenia would leave her susceptible to overwhelming falciparum malaria or bacterial sepsis, with a mortality rate approaching 50 % at 5 years [2]. In contrast, conservative management would leave the patient at an unacceptable risk of splenic rupture in a remote area without ready access to surgical services [2]. Management options explored included a non-operative approach with suppressive antimalarial therapy, less invasive procedures such as splenic artery embolisation (SAE), or surgical options including splenorenal shunt, total splenectomy, or PS. After multidisciplinary discussions between the surgeons, infectious disease physicians, haematologists, gastroenterologists, and anaesthetists, a consensus was reached; this patient was deemed suitable for PS.

### 2.4 The surgical intervention

**Fig. 2:**
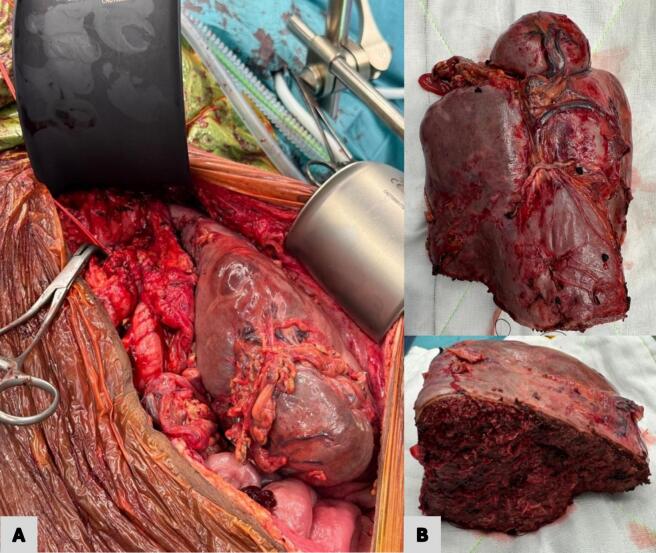
Intraoperative clinical photographs demonstrating massive splenomegaly (A) extending down to the left lower quadrant of the abdomen. (B) shows the transected 1049 g specimen following the partial splenectomy.

**Fig. 3:**
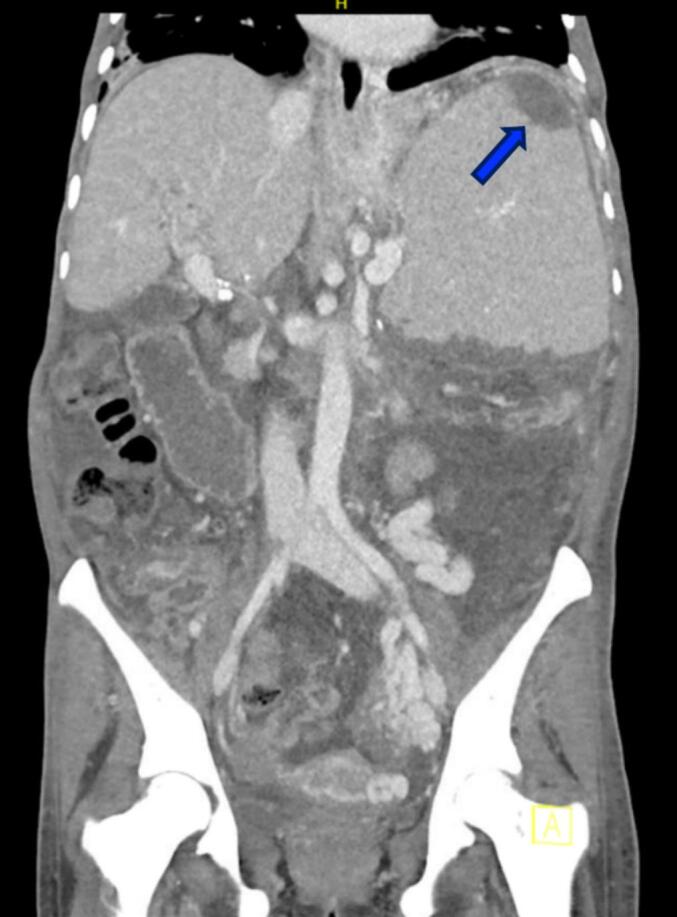
Post-operative CT of the abdomen and pelvis 5 days post open partial splenectomy assessing volume and supply to splenic remnant. A small focal infarct (blue arrow) is noted to the superior pole of the remnant spleen.

Asplenic vaccinations and an iron infusion were administered prior to surgery. The operative approach was a midline laparotomy, including xiphoidectomy, down to the pubis for optimal exposure. The splenic artery was carefully dissected in the lesser sac and controlled using a double vessiloop. Mobilisation of the spleen and control of the numerous varices were performed using a vessel-sealing device (LigaSure™ [15]), diathermy, and clips. The splenic hilum was dissected and the segmental arteries were slung. Intraoperative ultrasound was used to assess perfusion and determine the transection margins. The separate upper pole artery was subsequently preserved, and the hilum was stapled with 60 mm Echelon (vascular reload) [16]. A combination of ERBEJET® [17], LigaSure™, handheld diathermy and 60 mm Echelon was used for parenchymal transection and haemostasis. Further measures taken to promote haemostasis included the administration of 1 g IV tranexamic acid on induction and application of Floseal [18] to the lesser sac. Total estimated blood loss was 400 mL; therefore, 1 unit of PRBC was transfused intraoperatively along with 100 mL via cell saver. The resected PS specimen weighed 1049 g (Fig. 2). Histopathology confirmed splenomegaly with Gamna-Gandy bodies and acute infarction without evidence of neoplasia. This patient had an unremarkable postoperative recovery and was discharged from hospital day 32 of admission and day 9 following surgery. Both the routine postoperative CT scan to assess the splenic remnant (Fig. 3) and blood tests were reassuring just prior to discharge. She was well at her early clinic follow-up before returning home to Papua New Guinea. This patient remains on lifelong antimalarial suppressive therapy (100 mg oral doxycycline daily) per infectious disease specialist recommendations.

## 3 Discussion

The aim of therapeutic intervention for this patient was to address the burden of massive splenomegaly with infarcts, hypersplenism and the complications of portal hypertension with sinistral varices and recurrent UGIB. The other goal was to preserve splenic immunologic function, given that she would return to a poorly resourced malaria-endemic region.

SAE is a nonsurgical procedure used to manage portal hypertension associated with massive splenomegaly. A surgical lienorenal shunt may also be an option for patients with non-cirrhotic portal hypertension; however, this may be complicated by blockage or inadequacy of the shunt, resulting in persistent UGIB [19]. Although SAE is generally considered a safer alternative to surgery, it carries a high risk of complete infarction and abscess formation [20]. Given this patient would return to Papua New Guinea, both complications relating to SAE versus a conservative approach (meaning a risk of rupture and catastrophic haemorrhage leading to death) were carefully considered [21]. In the event of splenic infarction, abscess formation or splenic rupture, there would be limited ability to salvage the situation locally; therefore, it was deemed most appropriate to offer the patient surgery.

Current evidence only supports total splenectomy for two main groups of patients: the first being for those with hypersplenism and disabling symptoms, the second being those with massive malarial splenomegaly that is refractory to medical therapy [2]. At present, there are no documented cases detailing the treatment of hypersplenism or massive malarial splenomegaly with PS. According to the literature, indications for PS include cystic or hydatid disease, splenic tumour, haematological disease, such as chronic lymphocytic leukemia or hereditary spherocytosis, and splenic trauma [[22], [23], [24]]. This case report is hence unique in that it highlights the complex decision-making processes involved in managing this 40-year-old Papua New Guinean female with debilitating hypersplenism and previous HMSS. Moreover, it may encourage surgeons to consider PS and to document such cases where the burden of massive splenomegaly is too great, however, partial splenic preservation is preferred to maintain immunologic function.

## 4 Conclusion

This case report discusses the unique surgical management of complicated splenomegaly in a high-risk patient from a malaria-endemic country. While malaria is uncommon in Australia, the Northern Territory and Far North Queensland remain at risk of transmission from our neighbouring countries like Papua New Guinea. Clinicians practicing in tropical regions should thus maintain a higher level of suspicion for *P. falciparum* in patients with splenomegaly. Currently, there are no evidence-based decision-making guidelines when considering PS for hypersplenism and previous HMSS. Each individual case should therefore be carefully selected and the decision for PS made in conjunction with a multidisciplinary team of infectious disease specialists, haematologists, gastroenterologists, and surgeons. We are fortunate to report a case in which PS, which is a rarely performed operation, has had a positive impact on a patient with massive splenomegaly.

## Patient consent

Written informed consent was obtained from the patient for publication and any accompanying images. A copy of the written consent is available for review by the Editor-in-Chief of this journal on request.

## Ethical approval

Ethics approval is not required for case reports as there are not deemed to constitute research.

## Guarantor

The Guarantor is Dr Christian Bearsley, consultant General Surgeon of the Royal Australasian College of Surgeons and specialist in upper gastrointestinal surgery.

## Sources of funding

There are no sources of funding to disclose.

## Methods

This work has been reported in line with the SCARE criteria [25].

## Registration of research studies

N/A.

## Author contribution

Dr Minella Lalloz (myself) – corresponding author: study concept and write-up of full manuscript, including literature review, creation of figures, and references.

Dr Kate Swift– contributing author: editing of the manuscript, collation of intraoperative clinical photographs.

Dr Omar Mouline – contributing author: editing of the manuscript.

Dr Christian Beardsley – contributing author: editing of the manuscript, supervisor and guarantor for the case report.

## Declaration of competing interest

The authors declare that there are no conflicts of interest.
